# Work-family conflicts and self-reported work ability: cross-sectional findings in women with chronic musculoskeletal disorders

**DOI:** 10.1186/s12891-015-0515-4

**Published:** 2015-03-18

**Authors:** Matthias Bethge, Yvonne Borngräber

**Affiliations:** Institute of Social Medicine and Epidemiology, University of Lübeck, Ratzeburger Allee 160, 23562 Lübeck, Germany; Department of Rehabilitation Medicine, Hannover Medical School, Hannover, Germany; Institute of Medical Sociology and Rehabilitation Science, Charité - Universitätsmedizin Berlin, Berlin, Germany

**Keywords:** Family conflict, Employment, Rehabilitation, Disability, Musculoskeletal diseases

## Abstract

**Background:**

Under conditions of gender-specific division of paid employment and unpaid childcare and housework, rising employment of women increases the likelihood that they will be faced with work-family conflicts. As recent research indicates, such conflicts might also contribute to musculoskeletal disorders. However, research in patient samples is needed to clarify how important these conflicts are for relevant health-related measures of functioning (e.g., work ability). We therefore examined, in a sample of women with chronic musculoskeletal disorders, the indirect and direct associations between the indicators of work-family conflicts and self-reported work ability as well as whether the direct effects remained significant after adjustment for covariates.

**Methods:**

A cross-sectional questionnaire-based study was conducted. Participants were recruited from five rehabilitation centers. Work-family conflicts were assessed by four scales referring to time- and strain-based work interference with family (WIF) and family interference with work (FIW). Self-reported work ability was measured by the Work Ability Index. A confirmatory factor analysis was performed to approve the anticipated four-factor structure of the work-family conflict measure. Direct and indirect associations between work-family conflict indicators and self-reported work ability were examined by path model analysis. Multivariate regression models were performed to calculate adjusted estimators of the direct effects of strain-based WIF and FIW on work ability.

**Results:**

The study included 351 employed women. The confirmatory factor analysis provided support for the anticipated four-factor structure of the work-family conflict measure. The path model analysis identified direct effects of both strain-based scales on self-reported work ability. The time-based scales were indirectly associated with work ability via the strain-based scales. Adjusted regression analyses showed that a five-point increase in strain-based WIF or FIW was associated with a four- and two-point decrease in self-reported work ability, respectively. The standardized regression coefficients were β = 0.35 and β = 0.12.

**Conclusions:**

Our findings indicate that work-family conflicts are associated with poor work ability in female patients with chronic musculoskeletal disorders. However, longitudinal research is needed to establish a causal relationship. Better compatibility of work and family life might be an environmental facilitator of better rehabilitation outcomes in female patients with musculoskeletal disorders.

**Electronic supplementary material:**

The online version of this article (doi:10.1186/s12891-015-0515-4) contains supplementary material, which is available to authorized users.

## Background

In the European Union, the female employment rate rose to 58% over the past decade [1]. This development contributes to emancipation, better family income, social acknowledgement, self-esteem and self-realization. Under conditions of gender-specific division of paid employment and unpaid childcare and housework, however, it also increases the likelihood that women will experience psychological distress by organizing and harmonizing their work and family responsibilities [2]. These difficulties are consequences of work-family conflicts, a concept that was introduced by Greenhaus and Beutel as ‘a form of inter-role conflict in which the role pressures from work and family domains are mutually incompatible in some respect’ [3-7].

Research over the past decades has found robust evidence that work-family conflicts contribute to health-related problems and decreased quality of life. In Allen et al.’s [4] meta-analysis, work-family conflicts were negatively associated with job, life and material satisfaction and positively associated with general psychological strain, work- and family-related stress, somatic/physical symptoms, depression, and burnout. Similar findings are reported in a more recent meta-analysis by Amstad et al. [5]. These researchers also considered the reciprocal relationship of family and work, i.e., that the work role can interfere with the family role (work interference with family, WIF), as well as the family role can interfere with the work role (family interference with work, FIW). For both WIF and FIW, the strongest relationship was shown for domain-unspecific outcomes (e.g. life satisfaction, health problems, psychological strain and stress). Work-related outcomes (e.g. organizational citizenship behavior, work-related stress) were more strongly related to WIF, while family-related outcomes (e.g. material/family satisfaction and family-related stress) were more strongly associated with FIW.

While associations between work-family conflicts and general and mental health are well established, research on work-family conflicts as a potential cause or correlate of musculoskeletal disorders is still in its infancy. Some recent studies, however, suggest that work-family conflicts are associated with musculoskeletal pain [8-10]. For instance, Kim and colleagues [9] reported 2- to 3-times higher odds of musculoskeletal pain in persons with high work-family conflicts. Though these findings indicate the potential relevance of work-family conflicts as a risk factor for musculoskeletal disorders, further research is needed to clarify how important these conflicts are for relevant health-related measures of functioning (e.g., work ability). This is of particular importance as disability is increasingly recognized as the interaction of health state and environmental factors [11]. As work-family conflicts seem to be relatively stable over time [12], their potential impact on rehabilitation outcomes in patients with chronic disorders might limit the success of rehabilitation and return-to-work programs if this association is neglected.

Against this background, we investigated the association of work-family conflicts and work ability in a sample of women with chronic musculoskeletal disorders. We chose this outcome as improvement and restoration of work ability is a primary objective of rehabilitation programs in many Western countries [13]. Following Kelloway and colleagues [14] and others [15,16], we differentiated between both directions of role-conflicts (WIF and FIW), as well as time- and strain-based conflicts. More precisely, we explored indirect and direct associations between time- and strain-based WIF and FIW and self-reported work ability, and we examined whether the direct effects of strain-based WIF and FIW on work ability remained relevant after adjustment for important covariates.

## Methods

### Setting and participants

Participants were recruited from five inpatient rehabilitation centers for patients with chronic musculoskeletal disorders. In Germany, these services are provided by the German Pension Insurance in order to improve or to restore work ability and to prevent health-related early retirement. We included employed women at the beginning of their three-week rehabilitation programs. Rehabilitation was granted due to work ability restrictions related to musculoskeletal disorders. Data were collected through questionnaires. Age and diagnoses according to the International Classification of Diseases 10th revision (ICD-10) were extracted from the standardized rehabilitation discharge forms. Ethical approval was obtained from the Charité – Universitätsmedizin Berlin (EA1/049/11). Additional approval was gained from the data protection commissioner of the Federal German Pension Insurance.

### Measures

#### Work-Family Conflict Questionnaire

The German version the Work-Family Conflict Questionnaire (WFCQ) is based on the original version by Kelloway, Gottlieb and Barham [14] and consists of 22 items, which can be grouped into four subscales: time-based WIF (five items), strain-based WIF (six items), time-based FIW (five items) and strain-based FIW (six items). The translation procedure was guided by the recommendations of Beaton et al. [17] and involved five steps: first translation, design of a preliminary questionnaire, back translation, consent of a commission of experts and testing of the preliminary questionnaire.

The first translation was done by two people whose first language was the target language (German). Both researchers were familiar with the subject of the WFCQ. Additionally, the first phase was supported by the translation of an English teacher, acting as a naive element. She focused on the general comprehensibility of the items. This first translation procedure resulted in three versions. Throughout the course of designing a preliminary questionnaire, another German speaking researcher was consulted. Taking into account the original version of the WFCQ, all three translations were compared and a first version of the German WFCQ was prepared. Following this, two persons whose first language was the original language (English) independently retranslated the questionnaire. Both persons (Australian, US-American) were not familiar with the WFCQ and had no medical background. Based on all of the translations, all of the translators involved so far (commission of experts) created the final preliminary questionnaire.

This questionnaire version was preliminarily given to 154 female patients in order to test its linguistic comprehensibility. As there were no comments that indicated a further need for revision, this version was used in the current study. The original and the translated items are presented as Additional file 1. All of the items were rated using a four-point scale (1 = never; 4 = almost always). The total scores of the four subscales ranged from 5 to 20 points (time-based WIF and FIW) and from 6 to 25 points (strain-based WIF and FIW), respectively.

### Work Ability Index

Work ability was assessed using the German version of the Work Ability Index (WAI) questionnaire [16], a short self-report measure comprising the following subscores: (1) current work ability compared with lifetime best, (2) work ability in relation to the demands of the job, (3) number of current diseases diagnosed by a physician, (4) estimated work impairment due to disease, (5) sick leave during the past year, (6) own prognosis of work ability two years from now, and (7) mental resources.

The test-retest reliability of the WAI was found to be acceptable [18]. Moreover, several studies have confirmed that a poor WAI rating predicts productivity loss at work, retirement intentions, long-term sickness-related absences, early retirement and different indicators of need for rehabilitation [19-25]. Levels of work ability can be categorized as poor (7 to 27 points), moderate (28 to 36 points), good (37 to 43 points) and excellent (44 to 49 points).

### Covariates

We considered age, educational level (low vs. high, i.e. an enhanced lower secondary school certificate) and the primary rehabilitation diagnosis (M40–M54 according to the ICD-10 vs. other musculoskeletal diagnoses) as basic socio-demographic and medical data. For additional adjustments, we also assessed the responsibility for young children (at least one child ≤12 years vs. no children or all children >12 years) and the amount of working time (full-time vs. part-time).

### Data analyses

Descriptive statistics were used to characterize the recruited sample. In the case of continuous multi-item measures, Cronbach’s alpha was calculated to determine the internal consistency among items. Values >0.7 were regarded as satisfactory [26].

To check the factorial validity of the German WFCQ, a confirmatory factor analysis (CFA) was performed. This CFA tested whether the assumed four-factor model of the original WFCQ fit the data [27]. Several goodness-of-fit statistics were calculated to validate this assumption. First, the ratio of χ^2^ and degrees of freedom were obtained. Values less than three indicate a reasonable fit of the hypothesized model as compared to a saturated model [28]. Second, we checked the Goodness of Fit Index (GFI), the Comparative Fit Index (CFI), the Incremental Index of Fit (IFI) and the Tucker-Lewis Index (TLI). The four fit indices yield values ranging from zero to one, whereby values close to one are indicative of good fit and those greater than 0.90 or, even better, 0.95, generally indicate satisfactory fit [27]. Third, the root mean square error of approximation (RMSEA) was inspected. The RMSEA informs on the modeling of the covariance structure. Values less than 0.08 are indicative of good fit [27].

To determine the direct and indirect associations of the WFCQ scales and self-reported work ability as measured by the WAI, a path model analysis was performed. We assumed direct effects of both strain-based scales on the WAI and only indirect effects of the time-based scales, which were mediated by the corresponding strain-based scales. Bootstrapping with 2000 repetitions was performed to determine 95% confidence intervals of the direct and indirect effects. Moreover, modification indices were inspected to determine if additional paths would improve the fitting of the data. Goodness-of-fit statistics of the final model were examined as described above.

Finally, analyses of the assumed direct effects of strain-based WIF and FIW with self-reported work ability were complemented by a set of linear regression models. The first model considered both strain-based scales as explanatory variables. Educational level, age and primary rehabilitation diagnosis were added in the second model. The final model also included the amount of working time and the responsibility for young children.

Statistical differences were regarded as significant if the two-sided P value of a test was less than 0.05. AMOS 21 was used for the confirmatory factor analysis. All other calculations were performed with STATA SE 12.1.

## Results

### Participants

We included 351 employed women in our analyses. The number of recruited patients per center ranged from 33 to 105. Sample characteristics are shown in Table 1. Rehabilitation was mainly approved due to back pain-related ICD-10 diagnoses M40–M54. The mean age was 50.9 years (SD = 7.9). About 80% reported having attained at least an enhanced lower secondary school certificate, and 73% reported that they worked full time. Of the participants, 80% had children; 8% had at least one child aged 12 years or younger. The mean WAI score was approximately 28 points, indicating a rather severely restricted sample in terms of work ability. In the sample, 43% were categorized as women with poor work ability (7–27 points) and 44% as women with moderate work ability (28–36 points).

**Table 1:** **Sample characteristics**

	**n**	**mean (SD) or %**	**Cronbach’s alpha**
Age in years, mean (SD)	351	50.9 (7.9)	
Educational level	351		
% low		18.0	
% high		82.1	
ICD-10 diagnosis	351		
% M40–M54		84.6	
% M00–M25		10.5	
% other		4.8	
Children	351		
% at least one child ≤12 years		8.0	
% all children >12 years		71.8	
% none		20.2	
Employment	337		
% full-time		73.3	
% part-time		26.7	
Time-based WIF (5–20 points), mean (SD)	351	9.6 (2.9)	0.86
Strain-based WIF (6–25 points), mean (SD)	351	13.2 (3.1)	0.81
Time-based FIW (5–20 points), mean (SD)	351	7.1 (2.0)	0.68
Strain-based FIW (6–25 points), mean (SD)	351	9.3 (2.5)	0.83
Work Ability Index (7–49 points), mean (SD)	336	28.2 (7.7)	0.78
% poor (7–27 points)		42.9	
% moderate (28–36 points)		43.8	
% good/excellent (37–49 points)		13.4	

WIF, work interference with family; FIW, family interference with work; ICD-10, International Classification of Diseases 10th revision; SD, standard deviation.

### Factorial structure of the work-family conflict questionnaire

**Figure 1:**
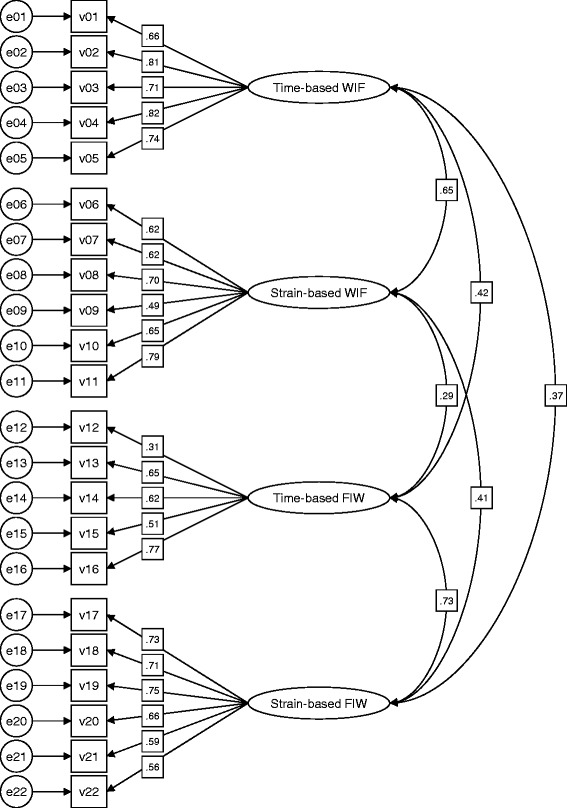
**Four-factor model of the WFCQ.** n = 351; χ^2^/df = 2.103; GFI = 0.902; CFI = 0.921; IFI = 0.922; TLI = 0.910; RMSEA = 0.056; WIF, work interference with family; FIW, family interference with work.

The standardized factor loadings and covariances of the CFA are depicted in Figure 1. The factor loadings of the observed variables were higher than 0.5, except for items 9 and 12. As expected, covariances were strongest between both FIW and both WIF factors. The anticipated four-factor model was confirmed by appropriate goodness-of-fit statistics. The ratio of χ^2^ and degrees of freedom were equal to 2.103, and the fit indices were consistently greater than 0.90 (GFI = 0.902; CFI = 0.921; IFI = 0.922; TLI = 0.910), indicating that the proposed four-factor model fit the sample’s data well. Additionally, the RMSEA was equal to 0.056, indicating adequate modeling of the covariance structure. The internal consistency of the scales was satisfactory, indicating that items which were used to form a scale were correlated as expected. For three scales, Cronbach’s alpha was even >0.8. Cronbach’s alpha was equal to 0.68 for the scale measuring time-based FIW (Table 1).

### Direct and indirect associations of work-family conflicts and work ability

**Figure 2:**
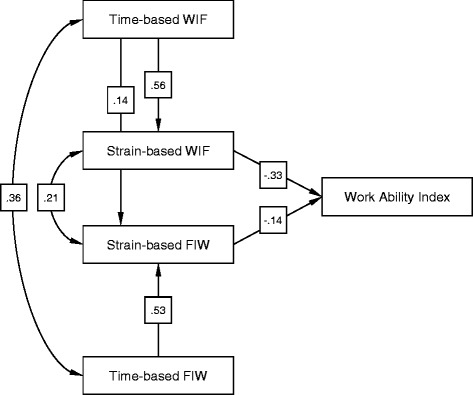
**Path model of indirect and direct associations of the WFCQ scales and self-reported work ability.** n = 336; χ^2^/df = 2.023; GFI = 0.993; CFI = 0.992; IFI = 0.992; TLI = 0.974; RMSEA = 0.055; WIF, work interference with family; FIW, family interference with work.

All dimensions of work-family conflicts were either directly or indirectly associated with self-reported work ability. The path model analysis identified direct effects of both strain-based scales on the WAI (strain-based WIF on WAI: β = −0.33; 95% CI: −0.42 to −0.21; strain-based FIW on WAI: β = -0.14; 95% CI: −0.24 to −0.04; Figure 2). There were no direct effects of both time-based scales on the WAI. However, as expected, there were significant indirect effects of both time-based scales, which were mediated by the strain-based scales (time-based WIF on WAI: β = -0.20; 95% CI: −0.27 to −0.14; time-based FIW on WAI: β = −0.07; 95% CI: −0.13 to −0.02). In addition to our hypothesized path model, we added an additional path from time-based WIF to strain-based FIW. Goodness-of-fit statistics of the final model were excellent.

### Adjusted analyses

All three regression models revealed significant associations between strain-based WIF and FIW and self-reported work-ability (Table 2). Adjustment for covariates hardly affected these associations. In the final model, a five-point increase in strain-based WIF or FIW was associated with a four-point and two-point decrease in self-reported work ability, respectively. The standardized regression coefficients were β = 0.35 and β = 0.12, indicating small to moderate associations between both strain-based scales and work ability. Self-reported work ability was better in women with a higher educational level. The responsibility for younger children was associated with a four-point decrease in self-reported work ability.

**Table 2:** **Cross-sectional associations of work-family conflicts and work ability**

	**Model 1**				**Model 2**				**Model 3**			
	**b**	**95% CI**	**p**	**β**	**b**	**95% CI**	**p**	**β**	**b**	**95% CI**	**p**	**β**
Strain-based WIF (per 5-point increase)	−4.1	−5.4; −2.8	<0.001	−0.34	−4.2	−5.5; −2.9	<0.001	−0.34	−4.3	−5.6; −3.0	<0.001	−0.35
Strain-based FIW (per 5-point increase)	−2.3	−3.9; −0.6	0.006	−0.15	−2.0	−3.6; −0.4	0.016	−0.13	−1.8	−3.4; −0.1	0.033	−0.12
Age (per 5-year increase)					0.3	−0.2; 0.8	0.213	0.06	0.1	−0.5; 0.6	0.812	0.01
Educational level: high					2.0	0.0; 4.1	0.047	0.10	2.4	0.4; 4.4	0.019	0.12
ICD-10 diagnosis: M40–M54					1.7	−0.4; 3.8	0.107	0.08	1.9	−0.2; 3.9	0.079	0.09
Employment: full-time									0.1	−1.6; 1.8	0.895	0.01
Children: at least one child ≤12 years									−4.0	−7.0; −1.0	0.009	−0.14

n = 327; b, unstandardized regression coefficient; CI, confidence interval; β, standardized regression coefficient; WIF, work interference with family; FIW, family interference with work; ICD-10, International Classification of Diseases 10th revision.

## Discussion

In our sample of female patients with chronic musculoskeletal disorders, the four-factor structure of the WFCQ was clearly approved and all four dimensions of work-family conflicts were either directly or indirectly associated with self-reported work ability. The strain-based scales affected work ability directly and the time-based scales affected work ability only indirectly via the strain-based scales. The direct effects of strain-based WIF and FIW on work ability were hardly affected by adjustment for several covariates. Though both strain-based scales were associated with self-reported work ability, the effect of strain-based WIF was twice as strong as the effect of strain-based FIW. Moreover, our use of continuous work-family conflict measures instead of categorized measures and the identified significant regression coefficients indicate a possible dose–response relationship between work-family conflicts and work ability.

The previous findings on the factorial structure of the WFCQ are not fully consistent. Two studies showed that the four-factor structure fit the data better than did any other structure (i.e. two or three factors) [14,29]. However, Noor [30] could only distinguish between WIF and FIW conflicts, and not between strain- and time-based conflicts. She therefore combined the two WIF scales in order to represent work-to-family conflicts and the two FIW scales in order to represent family-to-work conflicts. However, her sample was relatively small and consisted mainly of university employees. Our study on the German version supports the studies of Kelloway, Gottlieb and Barham [14] and Bragger et al. [29], as the four-factor structure fit the data well. Moreover, the scales were internally consistent and the scores of Cronbach’s alpha were similar to the ones that were reported in Kelloway, Gottlieb and Barham’s [14] original paper.

Additionally, Sanaz, Syaqirah and Khadijah [31] recently introduced a Malay version of the WFCQ. The researchers followed a similar translation procedure to the one we used and also replicated the questionnaire’s original four-factor structure (GFI = 0.93; CFI = 0.98; RMSEA = 0.036). Scores of Cronbach’s alpha above 0.75 also indicated that all four scales were of acceptable internal consistency. Further translations could enable cross-national studies in order to investigate if associations of work-family conflicts and health-related outcomes differ between countries or cultures.

Previous studies showed that work-family conflicts are associated with several health-related outcomes, including musculoskeletal disorders [4,5,8,9]. Our study of female patients with musculoskeletal disorders found that the strain-based scales of the WFCQ were also related to self-reported work ability. This finding is important as self-reported work ability, as measured with the WAI, has strong prognostic relevance for long-term health-related absences, disability pension and health-care utilization [19-24]. In addition, it is also increasingly recognized as a relevant outcome for evaluating the success of rehabilitation and occupational health programs [32,33].

Moreover, our findings also support the idea of Ilmarinen and others that work ability is not separate from life outside work, and that compatibility of work and family life is a major determinant of work ability [34]. As several studies [5,8], as well as our study, indicate that the effects of WIF are somewhat stronger than the effects of FIW, better compatibility of work and family life is not simply an individual or family challenge, but should be a major concern for employers’ human resource management teams.

From a practical point of view, it is significant that Noor’s [29] findings indicate that the associations of work-family conflicts and health are moderated by individual work salience. Individual-level interventions that help to reduce exaggerated work salience might create a buffer against the effects of work-family conflicts on health. Even more important, however, might be changes of the work culture in order to reduce work-family conflicts, as suggested by Bragger et al. [29]. These researchers interviewed 203 teachers in New Jersey and New York. Regression analyses indicated that work-family culture was associated with work-family conflicts, and the various forms of work-family conflicts were associated with organizational citizenship behavior. A supportive work-family culture seems to result in fewer work-family conflicts and might also prevent the deterioration of work ability.

A critical discussion of our findings needs to consider the following limitations. First, the recruited sample was restricted to women with chronic musculoskeletal disorders attending an inpatient rehabilitation program. Further research is needed to investigate the association of work-family conflicts and work ability in male patients. Contrary to earlier assumptions, recent findings indicate that both men’s and women’s health is negatively affected by work-family conflicts [7,35]. However, rising employment of women might increase the likelihood that women will perceive work-family conflicts. Though the consequences on health might be the same, work-family conflicts might be more frequent in women. Second, though the regression of work ability on the strain-based dimensions of work-family conflicts considered several covariates, we did not adjust our analyses for other known explanatory variables (e.g. type of work, effort-reward imbalance, and physical demands). Further research needs to clarify if the effect of work-family conflicts is redundant or complementary to the effects of other work stress factors. Third, the associations between the WFCQ scales with work ability are cross-sectional. Work-family conflicts might affect work ability. Conversely, deterioration of work ability may support the experience of work-family conflicts. We assume associations exist in both directions. However, to verify this hypothesis, longitudinal data and a cross-lagged panel analysis are required.

These limitations are balanced out by the following strengths. First, the translation procedure of the original WFCQ was guided by the recommendations of Beaton et al. [17]. This transparent and standardized procedure was chosen to minimize the risk of bias that may be introduced if an established measure is translated into another language. Second, the patients’ recruitment was done across multiple centers in order to strengthen generalizability. Third, we used path analysis to describe the associations of the four WFCQ scales with work ability in order to appropriately represent the anticipated direct and indirect associations.

## Conclusion

In conclusion, our findings are an initial hint that work-family conflicts might be a risk factor for poor work ability in female patients with musculoskeletal disorders. Further research, and above all, longitudinal research, is needed in order to generalize our findings. Stronger consideration of the work-related context in modern rehabilitation should avoid focusing solely on traditional occupational risk factors (e.g. conflicts with supervisors, monotonous work, and physical and psychological demands). Rehabilitation should broaden its perspective and better recognize the compatibility problems between employment and family work, and the different role expectations in work and family life. Moreover, supportive work-family cultures and better compatibility of work and family life might be environmental facilitators of better rehabilitation outcomes.

## Additional files

Additional file 1:
**Original and German items of the work-family conflict questionnaire.**

## Abbreviations

CFI: Comparative Fit Index
CI: Confidence interval
FIW: Family interference with work
GFI: Goodness of Fit Index
ICD-10: International Classification of Diseases 10th revision
IFI: Incremental Index of Fit
RMSEA: Root mean square error of approximation
SD: Standard deviation
SMD: Standardized mean differences
TLI: Tucker-Lewis Index
WAI: Work Ability Index
WFCQ: Work-Family Conflict Questionnaire
WIF: Work interference with family
