# Supplementary material for: Work-family conflicts and self-reported work ability: cross-sectional findings in women with chronic musculoskeletal disorders
Source: BMC Musculoskelet Disord. 2015 Mar 18;16:58. doi: 10.1186/s12891-015-0515-4 (PMC4373097; doi:10.1186/s12891-015-0515-4)
Supplement: Additional file 1: — Original and German items of the work-family conflict questionnaire. [file 12891_2015_515_MOESM1_ESM.docx]

Additional file 1: Original and German items of the Work-Family Conflict Questionnaire

|  | **Original items^*^** | **German items** |
| --- | --- | --- |
|  | *Time-based work interference with family* |  |
| 1. | I have to change plans with family members because of the demands of my job. | Aufgrund der Anforderungen in meinem Beruf muss ich Pläne, die ich mit meiner Familie gemacht habe, ändern. |
| 2. | Job demands keep me from spending the amount of time I would like with my family. | Die Anforderungen in meinem Beruf halten mich davon ab, so viel Zeit mit meiner Familie zu verbringen, wie ich möchte. |
| 3. | Job responsibilities make it difficult for me to get family chores/errands done. | Berufliche Verpflichtungen machen es mir schwer, Besorgungen für die Familie und den Haushalt zu erledigen. |
| 4. | To meet the demands of my job, I have to limit the number of things I do with family members. | Um meine beruflichen Anforderungen erfüllen zu können, muss ich die Zahl der Dinge, die ich mit meiner Familie mache, einschränken. |
| 5. | My job prevents me from attending appointments and special events for family members. | Mein Beruf hält mich davon ab, familiäre Verabredungen einzuhalten und besondere familiäre Anlässe wahrzunehmen. |
|  | *Strain-based work interference with family* |  |
| 6. | After work, I have little energy left for things I need to do at home. | Nach der Arbeit habe ich nur noch wenig Energie übrig, um Dinge zu Hause zu erledigen. |
| 7. | I think about work when I am at home. | Ich denke über die Arbeit nach, wenn ich zu Hause bin. |
| 8. | I do not listen to what people at home are saying because I am thinking about work. | Zu Hause höre ich meiner Familie nicht zu, weil ich an die Arbeit denke. |
| 9. | After work, I just need to be left alone for a while. | Nach der Arbeit möchte ich einfach eine Weile in Ruhe gelassen werden. |
| 10. | My job puts me in a bad mood at home. | Wegen meiner Arbeit habe ich zu Hause schlechte Laune. |
| 11. | The demands of my job make it hard for me to enjoy the time I spend with my family. | Aufgrund beruflicher Anforderungen fällt es mir schwer, die Zeit, die ich mit meiner Familie verbringe, zu genießen. |
|  | *Time-based family interference with work* |  |
| 12. | I would put in a longer workday if I had fewer family demands. | Wenn ich weniger familiäre Verpflichtungen hätte, würde ich länger auf der Arbeit bleiben. |
| 13. | My family demands interrupted my workday. | Meine familiären Verpflichtungen unterbrechen meinen Arbeitstag. |
| 14. | Family demands make it difficult for me to take on additional job responsibilities. | Familiäre Verpflichtungen machen es mir schwer, zusätzliche berufliche Aufgaben zu übernehmen. |
| 15. | I spend time at work making arrangements for family members. | Ich verbringe während der Arbeit Zeit damit, familiäre Angelegenheiten zu regeln. |
| 16. | Family demands make it difficult for me to have the work schedule I want. | Familiäre Verpflichtungen erschweren es mir, den Zeitplan für meine Arbeit so zu gestalten, wie ich es möchte. |
|  | *Strain-based family interference with work* |  |
| 17. | When I am at work, I am distracted by family demands. | Bei der Arbeit bin ich wegen familiärer Verpflichtungen abgelenkt. |
| 18. | Things going on in my family life make it hard for me to concentrate at work. | Aufgrund von Dingen, die in meiner Familie geschehen, fällt es mir schwer, mich bei der Arbeit zu konzentrieren. |
| 19. | Events at home make me tense and irritable on the job. | Durch Ereignisse zu Hause bin ich bei der Arbeit angespannt und gereizt. |
| 20. | Because of the demands I face at home, I am tired at work. | Wegen der Anforderungen zu Hause bin ich bei der Arbeit müde. |
| 21. | I spend time at work thinking about the things that I have to get done at home. | Bei der Arbeit verbringe ich Zeit damit, über Dinge nachzudenken, die ich zu Hause noch erledigen muss. |
| 22. | My family life puts me into a bad mood at work. | Aufgrund meines Familienlebens habe ich bei der Arbeit schlechte Laune. |

^*^ Kelloway EK, Gottlieb BH, Barham L: **The source, nature, and direction of work and family conflict: A longitudinal investigation**. *J Occup Health Psychol* 1999, **4**:337-346.
